# Examining the COVID-19 case growth rate due to visitor vs. local mobility in the United States using machine learning

**DOI:** 10.1038/s41598-022-16561-0

**Published:** 2022-07-19

**Authors:** Satya Katragadda, Ravi Teja Bhupatiraju, Vijay Raghavan, Ziad Ashkar, Raju Gottumukkala

**Affiliations:** Informatics Research Institute, University of Louisiana at Lafayette, Lafayette, USA

**Keywords:** Health policy, Data mining

## Abstract

Travel patterns and mobility affect the spread of infectious diseases like COVID-19. However, we do not know to what extent local vs. visitor mobility affects the growth in the number of cases. This study evaluates the impact of state-level local vs. visitor mobility in understanding the growth with respect to the number of cases for COVID spread in the United States between March 1, 2020, and December 31, 2020. Two metrics, namely local and visitor transmission risk, were extracted from mobility data to capture the transmission potential of COVID-19 through mobility. A combination of the three factors: the current number of cases, local transmission risk, and the visitor transmission risk, are used to model the future number of cases using various machine learning models. The factors that contribute to better forecast performance are the ones that impact the number of cases. The statistical significance of the forecasts is also evaluated using the Diebold–Mariano test. Finally, the performance of models is compared for three waves across all 50 states. The results show that visitor mobility significantly impacts the case growth by improving the prediction accuracy by 33.78%. We also observe that the impact of visitor mobility is more pronounced during the first peak, i.e., March–June 2020.

## Introduction

COVID-19 has spread rapidly around the world, nearing 389 million confirmed cases and more than 5.71 million deaths reported globally as of February 04, 2022 (John Hopkins University, 2020).

Many countries have tackled the spread of the pandemic through aggressive vaccination efforts, and other containment measures have limited the spread of the pandemic. However, it is important to understand the factors contributing to the spread of the virus. One crucial question that is still not answered is the degree of risk contribution of external visitors and if that is different than people traveling locally.

Earlier studies have examined travel patterns of populations to predict the spread of COVID-19^1–7^. Researchers used anonymized mobile phone data to track the commute and mobility patterns of the public^8–10^. Badr et al. found a strong correlation between the case growth rate of COVID-19 and the change in mobility patterns during the early phase of the pandemic, i.e., January 24th, 2020, to April 17, 2020, in the top 20 counties in the United States with the highest number of cases^11^. Other researchers also studied the relationship between the number of cases and the mobility patterns, including the stay-at-home orders^12–20^. Noland evaluated the impact of internal mobility on the case growth in a local region^21^. A more recent study looked at the impact of lockdowns on the mobility of the local population^22^.

Visitor mobility, specifically, has also been studied in different contexts. For instance, prior studies analyzed visitor traffic to various destinations to estimate potential COVID-19 risk exposure^23–25^. However, no studies examined the difference between travel patterns of local traffic and inbound traffic. Linka et al. used a global network mobility model with a local epidemiology model to simulate and predict the COVID-19 outbreak across Europe^19^. The authors show that mobility networks of air travel can predict the global diffusion patterns of a pandemic and that unconstrained mobility accelerated the spread of COVID-19 in Europe, using an incubation period of 2–6 days and an infectious period of 3–18 days. Other studies from Wuhan used real-time mobility data combined with the detailed travel history to explain the spread of COVID across China^26^. The analysis showed that the outbound traffic from Wuhan could explain the number of COVID-19 cases in China. Their analysis also showed that travel restrictions for inbound traffic and the local mitigation strategies reduced the transmission of the virus.

In a study evaluating the effect of mobility restriction in limiting COVID-19 spread, using zip code data for Atlanta, Boston, Chicago, New York (NYC), and Philadelphia, the authors estimated that total COVID-19 cases per capita decreased on average by approximately 20% for every 10% fall in mobility between February and May 2020^17^. In another study, the correlation between the COVID-19 growth rate and travel distance decay rate and dwell time at home change rate was − 0.586 (95% CI − 0.742 to − 0.370) and 0.526 (95% CI 0.293–0.700), respectively. Increases in state-specific doubling time of total cases ranged from 6.86 to 30.29 days after social distancing orders were put in place^4^. Another analysis across counties in the US showed that the adoption of government-imposed social distancing measures reduced the daily growth rate of confirmed COVID-19 cases by 5.4 percentage points after 1–5 days, 6.8 percentage points after 6–10 days, 8.2 percentage points after 11–15 days, and 9.1 percentage points after 16–20 days^13^. IA recent European study showed that internal mobility is more important than mobility across provinces to control COVID-19, and the typical lagged positive effect of reduced human mobility in reducing excess deaths is around 14–20 days. Similarly, Linka et al. evaluated the impact of global mobility using air travel and local mobility across various countries in the European Union. Their results show a maximal correlation between driving mobility and disease dynamics with a time lag of 14.6 ± 5.6 days.

Similarly, in the United States, various states, counties, and cities imposed restrictions on travel locally and from people traveling outside. For example, early in the pandemic, non-essential businesses were closed to curb the spread of the virus. Later in the pandemic, restrictions were imposed for visitors traveling from high-risk regions. For example, the United States restricted travel from various countries with a high number of COVID cases. Similarly, states like Illinois and New York mandated vaccination proofs for travelers from states with an increased number of cases during the Delta variant. A question that has not been explored is whether the impact of visitor mobility is different than local mobility and which of the either contributes more to the spread of the virus.

In this study, we examine the impact on the daily number of COVID cases resulting from local mobility and visitor mobility for all the states in the United States. In the United States, most public health decisions are made at the State level. In practice, the impact of mobility across state borders is limited (i.e., the number of people crossing state boundaries) compared to mobility between counties, especially in counties in the same metro region. Also, given the number of counties in the United States and their variability in size, population, and socioeconomic factors, it is challenging to arrive at generalizable conclusions. Therefore, we consider the state-level granularity for this analysis. We consider two different variables to capture the infection propagation risk from infected people traveling and transmitting the virus within and across state boundaries, namely the local transmission risk (due to local mobility) and the visitor transmission risk (due to visitor mobility). We evaluate the impact of these variables to predict the case growth using various machine learning models. Assuming all the other variables like social distancing measure, mask mandates, and the local number of cases are similar, we evaluate which combination of the cases, local, and visitor mobility are more accurate at predicting the future number of cases. The more accurate the models are, the higher the impact of the variables included in the model on the target variable (the case growth)^27^. This study analyzes the mobility data both within and among all 50 states and the number of new cases per day in the United States.

## Methods

### Data collection

#### Infection data

The confirmed cases data was retrieved from the Corona Data Scraper open-source project^28^, which provides county-level data on the number of new cases per day. We aggregated the number of daily new cases to a state-level between March 1, 2020, and December 31, 2020.

#### Mobility data

State-level mobility datasets and metrics were provided by SafeGraph^29^. SafeGraph provides aggregated trip information obtained from anonymized mobile device locations at a census tract level. The intra-state (or local) trips represent the trips taken by individuals starting and ending within the same state (i.e., state boundary). The inter-state (or visitor) trips are those where the origin and destination are in different states (i.e., origin in one state and destination in a different state). This data was collected for all the trips made between March 1, 2020, and December 31, 2020.

### Approach

To measure the impact of mobility (both local and visitor), we model the number of cases at a particular location based on the historical number of cases and the transmission of infection based on mobility. The risk of mobility associated with COVID transmission is calculated as a product of traffic flow and the number of cases per capita at origin^30^. Therefore, the local and visitor transmission risk reflects the number of infected individuals traveling from origin to destination, assuming uniform transmission. It has been established in the literature that a higher accuracy when a new feature is introduced into a machine learning model indicates that the particular feature is an important predictor for the target variable^27^. For a state *i*, we introduce three features, the current number of cases ($C_i$), local transmission risk ($LT_i$), and visitor transmission risk ($VT_i$), to predict the future number of cases in a particular state. Higher forecasting accuracy when using visitor transmission risk means that it impacts the number of future cases. The prediction model is built using both linear and non-linear regression-based machine learning approaches and a combination of the three features noted earlier. More information about the features and the machine learning methods is provided below:

#### Number of cases

The aggregated new cases from the previous 14 days are used to forecast the number of cases for the next 14 days; earlier studies have shown that the virus incubation period is about 14 days^31^.

#### Local transmission risk

The local transmission risk represents the transmission potential of the virus based on the recent number of cases per capita (which represents local case incidence) and the mobility both at the local level. The local transmission coefficient *LT* for a spatial region *i* is calculated using the formula:

1 $$\begin{aligned} LT_i = M_{i,i} \times C_i \end{aligned}$$

where $M_{i,i}$ represents the number of trips where the origin and destination of the trips fall within the region *i*. The cases per 100,000 people at location *i*, which we denote as $C_i$.

#### Visitor transmission risk

**Figure 1:**
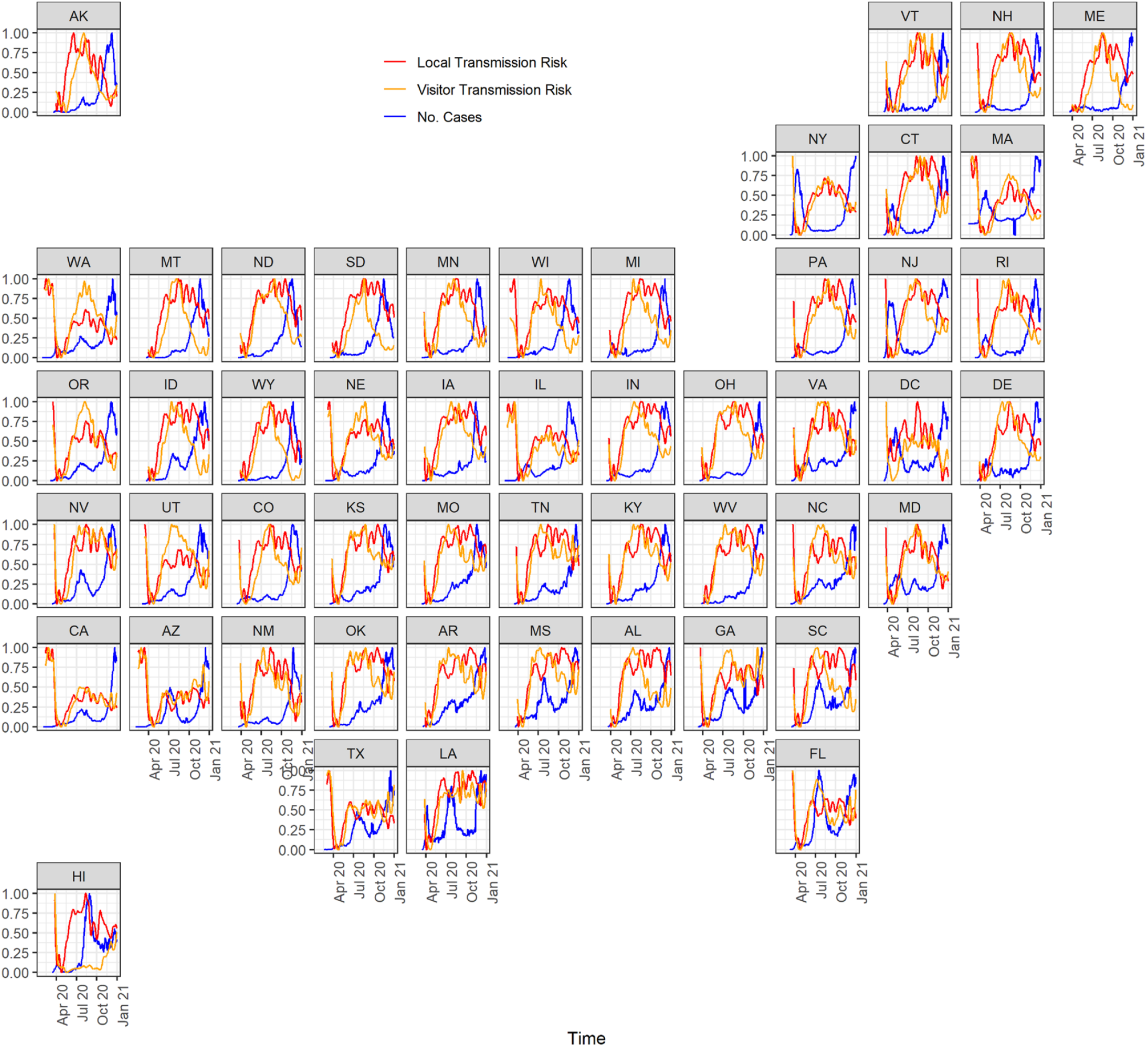
The timeseries of the cases per capita, local transmission risk, and visitor transmission risk for various states in the United States.

The visitor transmission risk represents the transmission potential of the virus based on the recent number of cases per capita at the visitor origin. The visitor transmission risk *VT* at a location *i* can be calculated using:

2 $$\begin{aligned} VT_i = \sum _{j=0}^{n}M_{j,i} \times C_j \end{aligned}$$

where $M_{j,i}$ represents the number of trips that originate at *j* and end at location *i* and $j \ne i$. The cases per capita at location *j* are represented by $C_j$. These three measures are illustrated in Fig. 1.

#### Relationship between number of cases and transmission risk

The relationship between the number of cases and mobility is formulated using the below regression equations, where number of cases is the dependent variable, and the mobility related risks are the independent variables.

3 $$\begin{aligned} C_{i} = h[C_{i-14},\ LT_{i-14},\ VT_{i-14}, \theta _{C_{i-14}}, \theta _{LT_{i-14}}, \theta _{VT_{i-14}} ] + E_i \end{aligned}$$

where $C_{i-14}$, $LT_{i-14}$, $VT_{i-14}$ refer to the inputs (or independent variables) to the model, the current number of cases, local transmission risk, and the visitor transmission risk lagged by 14 days, respectively. The dependent or the predicted variable is the current number of cases. The three parameters $\theta _{C_{i-14}}, \theta _{LT_{i-14}}, \theta _{VT_{i-14}}$ are parameters to be estimated. The function h is deduced using a machine learning approach, and the impact of historical cases and no transmission risk, historical cases and local transmission risk, historical cases and visitor transmission risk, and finally, historical cases, local and visitor transmission risk, are used to understand the impact on the future number of cases. Comparing the accuracy of different models and evaluating the statistical significance provides insights into which variables have a higher impact on the case growth.

### Machine learning methods

Earlier work on studying mobility and its impact on the number of COVID cases identifies the relationship as non-linear^18^. Machine learning models can capture both linear and non-linear relationships between different variables. The abundance of the COVID data-case data and mobility patterns, enables us to identify complex relationship patterns. This study used popular machine learning methods: Linear Regression, Support Vector Regression, K-Nearest Neighbor Regression, Multilayer Perceptron, Random Forest Regression, and eXtreme Gradient Boost (XGBoost) Regression to forecast the number of cases. The linear regression model is linear, the other models are non-linear, where the Support Vector Regression is a support vector machine based approach, K-Nearest Neighbor is a similarity based approach, Multilayer perceptron is a neural network based approach, and finally, the random forest and XGBoost are decision tree based. These models consider the historical number of cases, local transmission risk, and visitor transmission risk when forecasting the future number of cases.

### Evaluation criteria

The predictive performance of the proposed approach for each of the stations is compared using the following two metrics: mean absolute percentage error (MAPE) measures the average percent of absolute deviation between actual and forecasted values.

4 $$\begin{aligned} MAPE = \frac{1}{N} \sum \frac{\left| A-P \right| }{A} \times 100 \end{aligned}$$

Root mean squared error (RMSE) captures the square root of the average of squares of the difference between actual and forecasted values.

5 $$\begin{aligned} RMSE = \sqrt{\frac{1}{N}\sum (A-P)^2} \end{aligned}$$

where *N* is the number of test samples, *A* is the actual value, and *P* is its predicted value. For each technique, we evaluate the accuracy of prediction with and without the visitor transmission risk.

Diebold–Mariano test (DM-test) is used to evaluate the significance of the predictions of the two models^32^. The models use the forecasted number of cases generated with and without using the visitor transmission for each of the three machine learning approaches. The null hypothesis of the DM-test is that the two forecasts have similar forecast accuracy. The alternative or rejection of the null hypothesis is that the two forecasts have significantly different forecasting accuracy, i.e., the forecasts are not similar using the two models.

The results for each of the models are evaluated using 10-fold cross-validation at a state level to ensure that the data is not overfitting. The data for each state is extracted and split into 10 equal subsets. The data is trained using the 9 subsets and evaluated on the 10 for each of the subsets. The reported results are the average MAPE and RMSE for all the 10 folds.

**Table 1:** Comparison of MAPE to forecast the number of cases with and without visitor transmission risk for various machine learning models.

Model	No mobility	Local transmission risk only	Visitor transmission risk only	Local and visitor transmission risk
Linear regression	0.512 ± 0.212	**0.464 ± 0.188**	0.535 ± 0.268	0.497 ± 0.227
KNN regression	0.341 ± 0.301	0.338 ± 0.143	0.329 ± 0.214	**0.317 ± 0.149**
Support vector regression	0.758 ± 0.279	0.752 ± 0.253	**0.771 ± 0.324**	0.779 ± 0.316
Multilayer perceptron	0.398 ± 0.126	0.385 ± 0.114	0.374 ± 0.13	**0.356 ± 0.109**
Random forest regression	0.334 ± 0.123	0.327 ± 0.121	0.309 ± 0.106	**0.305 ± 0.105**
XGBoost	0.243 ± 0.117	0.222 ± 0.098	0.17 ± 0.067	**0.168 ± 0.067**

Best scenario for each machine learning approach is highlighted in [bold].

**Table 2:** Comparison of RMSE to forecast the number of cases with and without visitor transmission risk for various machine learning models.

Model	No mobility	Local transmission risk only	Visitor transmission risk only	Local and visitor risk
Linear regression	0.108 ± 0.027	0.105 ± 0.026	0.102 ± 0.025	**0.099 ± 0.026**
KNN regression	0.089 ± 0.032	0.084 ± 0.027	0.08 ± 0.031	**0.071 ± 0.027**
Support vector regression	0.1 ± 0.028	0.096 ± 0.027	0.092 ± 0.025	**0.089 ± 0.024**
Multilayer perceptron	0.083 ± 0.34	0.079 ± 0.031	0.07 ± 0.025	**0.068 ± 0.023**
Random forest regression	0.075 ± 0.036	0.074 ± 0.034	0.063 ± 0.026	**0.062 ± 0.255**
XGBoost	0.083 ± 0.045	0.075 ± 0.038	0.055 ± 0.023	**0.055 ± 0.024**

Best scenario for each machine learning approach is highlighted in [bold].

## Results

Tables 1 and 2 compare the machine learning forecasts with and without the inclusion of visitor mobility and local mobility. We compare the performance of the three machine learning models (XGBoost, Linear Regression, and Random Forecast) using the MAPE and RMSE. The results show that the MAPE of the forecasted cases is higher when both the local and visitor transmission risk is taken into account for the top 4 of the 6 models, and the RMSE of the forecasted number of cases is higher for all the models. We also evaluated the forecasting capacity of the models when the mask mandates and the social distancing guidelines were also used as features in the machine learning models to forecast the number of cases; we did not notice a significant improvement in the forecasting capability. In addition, a DM test was performed to evaluate the significance of forecasts when visitor mobility is included in the model.

Table 1 shows the MAPE of machine learning models for the complete duration (i.e., March 2020–December 2020). We observe that the MAPE of the best performing model (i.e., XGBoost) has a MAPE of 16.8% when using visitor mobility compared to 22.2% when just the local transmission risk is taken into account. The linear regression model performs better when using local transmission risk than combined local and visitor transmission risk. However, Table 2 shows that the RMSE is lower when using the combination of local and visitor transmission risk than local transmission risk. In addition, the RMSE is lower when local and visitor mobility is used for all three models.

The significance of the results is evaluated using the Diebold–Mariano score that evaluates the null hypothesis that both the forecasts are the same. The p value shows that the null hypothesis is rejected, and the difference in forecasts is statistically significant. The DM scores for the XGBoost, linear regression, and random forest are 8.7 (p = 0.2), 2.66 (p = 0.04), and 8.26 (p = 0.04) respectively. The MAPE and RMSE on the state-level data in Table 5 show that the inclusion of external mobility leads to better forecasts for all 50 states in the United States.

**Table 3:** Comparison of forecasting performance using MAPE with and without local and visitor transmission risk for three waves using various machine learning models.

Model	No mobility	Local transmission risk only	Visitor transmission risk only	Local and visitor transmission risk
**First wave (March–June)**				
XGBoost	0.376	0.308	0.169	**0.174**
Linear regression	1.064	1.029	1.094	**0.938**
Random forest	0.422	0.409	0.329	**0.327**
**Second wave (July–September)**				
XGBoost	1.038	0.999	0.885	**0.834**
Linear regression	1.411	1.35	1.251	**1.208**
Random forest	1.023	1.007	0.887	**0.875**
**Third wave (October–December)**				
XGBoost	0.469	0.492	0.352	**0.341**
Linear regression	1.401	1.369	1.174	**1.142**
Random forest	0.498	0.508	0.369	**0.359**

Best scenario for each machine learning approach is highlighted in [bold].

**Table 4:** Comparison of forecasting performance using RMSE with and without local and visitor transmission risk for three waves using various machine learning models.

Model	No mobility	Local transmission risk only	Visitor transmission risk only	Local and visitor transmission risk
**First wave (March–June)**				
XGBoost	0.145	0.113	0.074	**0.072**
Linear regression	0.161	0.143	0.145	**0.128**
Random forest	0.123	0.113	0.083	**0.082**
**Second wave (July–September)**				
XGBoost	0.223	0.201	0.164	**0.158**
Linear regression	0.207	0.199	0.185	**0.18**
Random forest	0.188	0.183	0.154	**0.153**
**Third wave (October–December)**				
XGBoost	0.118	0.109	**0.08**	**0.08**
Linear regression	0.183	0.183	0.165	**0.164**
Random forest	0.102	0.101	**0.08**	**0.08**

Best scenario for each machine learning approach is highlighted in [bold].

We make similar observations when the data is separated into three waves (Tables 3, 4) to show the performance of the model using the top two machine learning approaches along with linear regression as a baseline. The MAPE and RMSE for all the three approaches report lower MAPE and RMSE when visitor transmission risk is included in the model generation compared to just the local mobility risk. The inclusion of the visitor transmission risk improves the MAPE of the forecast by about 57.19% on average across all states for the three waves. During the first wave, the external mobility decreases MAPE by 110%, with the percentage error at 17.14 ± 0.28% and 30.81 ± 0.48% with external and only local transmission coefficients, respectively. Similarly, the improvement for MAPE for the second and third waves is 34.23% and 26.17%, respectively.

**Table 5:** The importance of various features to calculate the future number of cases using a random forest regression.

Feature	All waves	First wave	Second wave	Third wave
Number of cases	0.646	0.611	0.652	0.731
Visitor transmission risk	0.249	0.303	0.237	0.241
Local transmission risk	0.105	0.086	0.111	0.028

Table 5 shows the importance of each feature when forecasting the number of future cases using a random forest regressor. The importance of each feature is calculated using an estimator based on the increase in error when the particular feature is not considered. The results show that the current number of cases has the highest impact on the number of cases. However, the visitor transmission potential is more important than the local transmission risk for all the waves of the pandemic. The visitor transmission risk is considered twice as important as the local transmission risk.

These results show that the non-linear models can accurately predict the number of new cases in the future with high accuracy when considering the visitor mobility risk along with the local mobility risk and the current number of cases. With the rest of the factors like social distancing, mask mandates, and vaccination status constant, visitor mobility is a significant factor in determining the number of cases. We also note that the impact of local and visitor mobility risk is not consistent across the three waves of the pandemic. During the first and the third waves, mobility has a higher impact on the number of cases compared to the second wave. The forecasting performance for each state using XGBoost for the three waves of the pandemic in 2020 is presented in Table 6.

**Table 6:** The accuracy for each state for case forecasting using visitor and local, and just local transmission risk using XGBoost.

FIPS	All waves				First wave				Second wave				Third wave			
	MAPE		RMSE		MAPE		RMSE		MAPE		RMSE		MAPE		RMSE	
	Visitor + local	Local	Visitor + local	Local	Visitor + local	Local	Visitor + local	Local	Visitor + local	Local	Visitor + local	Local	Visitor + local	Local	Visitor + local	Local
1	0.14	0.17	0.06	0.07	0.08	0.12	0.03	0.04	1.7	1.78	0.24	0.33	0.64	0.63	0.09	0.1
2	0.18	0.22	0.05	0.06	0.25	0.64	0.04	0.1	0.42	0.43	0.14	0.14	0.28	0.22	0.09	0.11
4	0.26	0.4	0.07	0.14	0.06	0.09	0.02	0.03	0.33	0.28	0.07	0.08	0.13	0.11	0.03	0.02
5	0.1	0.11	0.03	0.03	0.09	0.16	0.03	0.04	1.3	1.83	0.21	0.28	0.42	0.38	0.07	0.07
6	0.13	0.19	0.03	0.05	0.05	0.05	0.02	0.02	0.37	0.62	0.17	0.28	0.22	0.2	0.04	0.02
8	0.14	0.2	0.04	0.07	0.1	0.15	0.05	0.08	1.04	1.28	0.2	0.23	0.23	0.25	0.09	0.14
9	0.35	0.4	0.08	0.09	0.56	0.49	0.22	0.21	0.85	0.9	0.1	0.12	0.14	0.11	0.06	0.06
10	0.2	0.23	0.04	0.05	0.27	0.33	0.15	0.17	0.83	0.88	0.16	0.21	0.33	0.37	0.04	0.04
12	0.27	0.3	0.12	0.14	0.13	0.13	0.03	0.02	0.4	0.56	0.16	0.26	0.22	0.31	0.04	0.04
13	0.14	0.19	0.06	0.08	0.08	0.12	0.03	0.05	1.34	1.81	0.17	0.29	0.46	1.05	0.08	0.12
15	0.31	0.52	0.06	0.13	0.81	1.97	0.13	0.3	0.22	0.44	0.06	0.25	0.73	1.71	0.11	0.26
16	0.19	0.27	0.05	0.06	0.2	0.5	0.05	0.11	1.93	2.05	0.28	0.31	0.19	0.2	0.06	0.07
17	0.12	0.21	0.04	0.06	0.08	0.28	0.05	0.17	0.25	0.11	0.06	0.07	0.23	0.21	0.09	0.1
18	0.09	0.13	0.03	0.03	0.07	0.09	0.04	0.05	0.45	0.25	0.13	0.13	0.15	0.11	0.06	0.05
19	0.16	0.24	0.05	0.13	0.13	0.13	0.1	0.09	1.56	1.98	0.27	0.28	0.46	0.82	0.14	0.33
20	0.13	0.2	0.04	0.04	0.17	0.4	0.08	0.2	1.66	1.36	0.23	0.21	0.33	0.22	0.08	0.06
21	0.09	0.09	0.03	0.03	0.08	0.14	0.05	0.1	0.42	0.2	0.09	0.08	0.2	0.15	0.07	0.06
22	0.3	0.34	0.14	0.16	0.15	0.31	0.06	0.14	0.73	0.81	0.26	0.29	1.7	1.76	0.17	0.19
23	0.14	0.21	0.05	0.06	0.15	0.27	0.09	0.1	0.7	0.82	0.14	0.23	0.36	0.66	0.09	0.1
24	0.16	0.21	0.06	0.08	0.13	0.19	0.08	0.12	0.94	0.99	0.26	0.29	0.2	0.11	0.04	0.03
25	0.26	0.35	0.06	0.09	0.43	0.54	0.18	0.2	0.58	0.52	0.14	0.17	0.18	0.14	0.06	0.08
26	0.13	0.23	0.06	0.08	0.14	0.29	0.06	0.14	0.24	0.27	0.08	0.09	0.3	0.31	0.13	0.17
27	0.16	0.19	0.07	0.1	0.11	0.15	0.08	0.12	0.51	0.47	0.17	0.17	0.46	0.47	0.17	0.22
28	0.14	0.21	0.07	0.1	0.08	0.18	0.05	0.11	1.43	1.87	0.14	0.31	0.31	0.29	0.07	0.08
29	0.09	0.09	0.04	0.04	0.11	0.13	0.05	0.05	0.22	0.18	0.06	0.08	0.35	0.3	0.11	0.11
30	0.16	0.25	0.04	0.1	0.43	1.73	0.04	0.13	0.32	0.32	0.08	0.07	0.59	1.66	0.11	0.29
31	0.15	0.18	0.08	0.09	0.12	0.15	0.07	0.1	0.32	0.3	0.07	0.07	0.47	0.41	0.23	0.24
32	0.21	0.21	0.07	0.07	0.09	0.1	0.03	0.03	0.96	0.95	0.24	0.28	0.16	0.12	0.05	0.03
33	0.15	0.16	0.03	0.03	0.18	0.18	0.09	0.08	0.46	0.76	0.11	0.15	0.13	0.14	0.04	0.04
34	0.26	0.42	0.11	0.15	0.41	0.54	0.23	0.27	1.13	1.56	0.13	0.18	0.2	0.12	0.04	0.02
35	0.19	0.22	0.04	0.05	0.08	0.16	0.04	0.11	1.32	1.52	0.24	0.26	0.17	0.13	0.07	0.09
36	0.2	0.42	0.06	0.17	0.19	0.61	0.07	0.27	0.41	0.68	0.07	0.1	0.16	0.1	0.03	0.02
37	0.09	0.09	0.03	0.03	0.05	0.05	0.02	0.02	2.09	2.04	0.32	0.32	0.22	0.21	0.05	0.05
38	0.17	0.26	0.05	0.14	0.16	0.21	0.1	0.11	0.13	0.12	0.04	0.04	0.99	3.34	0.13	0.33
39	0.11	0.12	0.03	0.03	0.07	0.17	0.04	0.12	1.1	1.69	0.2	0.23	0.13	0.1	0.04	0.04
40	0.11	0.13	0.05	0.05	0.11	0.16	0.04	0.06	0.43	0.66	0.14	0.25	0.8	0.66	0.13	0.15
41	0.11	0.12	0.03	0.03	0.12	0.16	0.04	0.06	2.05	1.83	0.33	0.34	0.24	0.19	0.05	0.05
42	0.15	0.16	0.02	0.02	0.17	0.22	0.13	0.17	0.82	1.34	0.12	0.17	0.12	0.1	0.03	0.03
44	0.32	0.32	0.06	0.06	0.42	0.47	0.21	0.24	0.26	0.35	0.11	0.11	0.13	0.1	0.05	0.05
45	0.16	0.18	0.08	0.1	0.07	0.1	0.02	0.02	0.7	1.07	0.17	0.26	0.49	0.65	0.08	0.1
46	0.13	0.2	0.04	0.1	0.11	0.15	0.06	0.08	0.31	0.34	0.08	0.08	0.71	2.13	0.11	0.28
47	0.13	0.14	0.05	0.05	0.08	0.13	0.03	0.04	0.97	1.26	0.18	0.3	0.29	0.35	0.09	0.1
48	0.15	0.18	0.07	0.08	0.08	0.09	0.02	0.02	1.2	1.28	0.24	0.31	0.61	0.84	0.08	0.1
49	0.1	0.15	0.03	0.05	0.08	0.1	0.03	0.04	0.78	2.97	0.09	0.26	0.19	0.19	0.08	0.09
50	0.32	0.44	0.04	0.08	0.36	0.86	0.1	0.27	1.07	1.2	0.2	0.21	0.13	0.14	0.04	0.05
51	0.1	0.13	0.03	0.04	0.12	0.15	0.07	0.1	0.44	0.51	0.16	0.22	0.2	0.17	0.04	0.04
53	0.16	0.19	0.06	0.06	0.1	0.17	0.04	0.09	1.65	1.49	0.27	0.27	0.2	0.14	0.06	0.05
54	0.12	0.14	0.02	0.02	0.26	0.46	0.11	0.16	0.52	0.92	0.1	0.16	0.15	0.12	0.03	0.03
55	0.11	0.17	0.04	0.1	0.13	0.17	0.08	0.1	1.03	1.22	0.11	0.12	0.47	0.88	0.09	0.29
56	0.19	0.21	0.09	0.1	0.2	0.27	0.11	0.15	0.81	0.86	0.1	0.12	0.44	0.5	0.19	0.25

**Figure 2:**
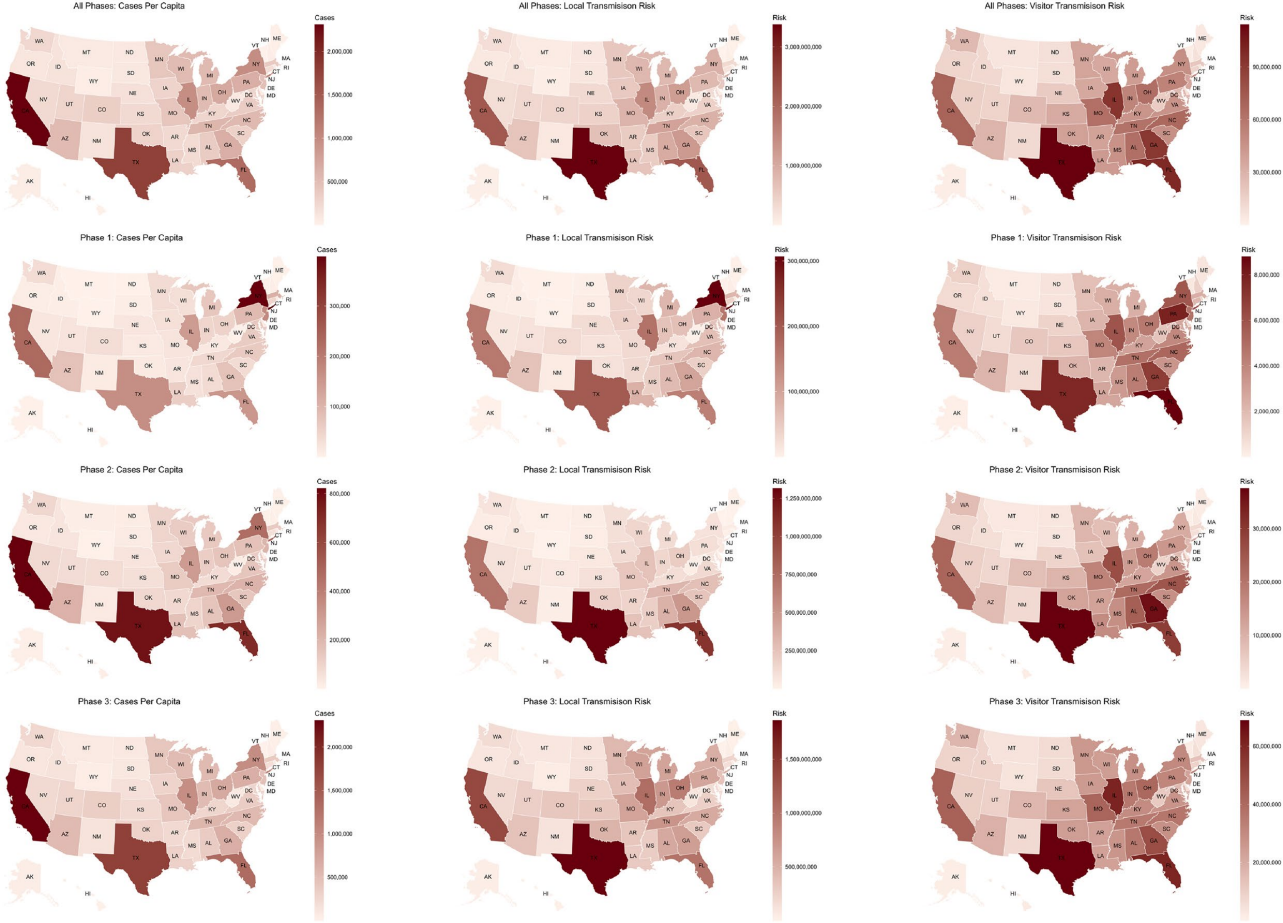
Distribution of the number of cases, local transmission risk, and visitor transmission risk to various states across all phases (March–December), phase 1 (March–June), phase 2 (July–August), and phase 3 (October–December). Certain states with a high number of cases have high local transmission risk, whereas others have high visitor transmission risk, where risk is imported from outside the state boundaries. All maps generated using urbnmapr^33^.

## Discussion of results

Figure 2 shows the cumulative number of cases per capita, local transmission risk, and visitor transmission risk for each of the states in the United States for the entire pandemic and the three phases of the pandemic. We observe that certain states have a lower local transmission risk and a higher visitor transmission risk. For example, during the second phase of the pandemic, in states like Illinois and Georgia, the local transmission risk is much lower than the transmission risk posed by travelers from other states to Illinois. Similarly, for states like New York in phase 1, the local transmission risk is higher than the visitor transmission risk. There are also variations between the interplay of local and visitor transmission risks for different phases of the pandemic. The first phase is primarily driven by local mobility, and the other two phases are a combination of local and visitor mobility.

**Figure 3:**
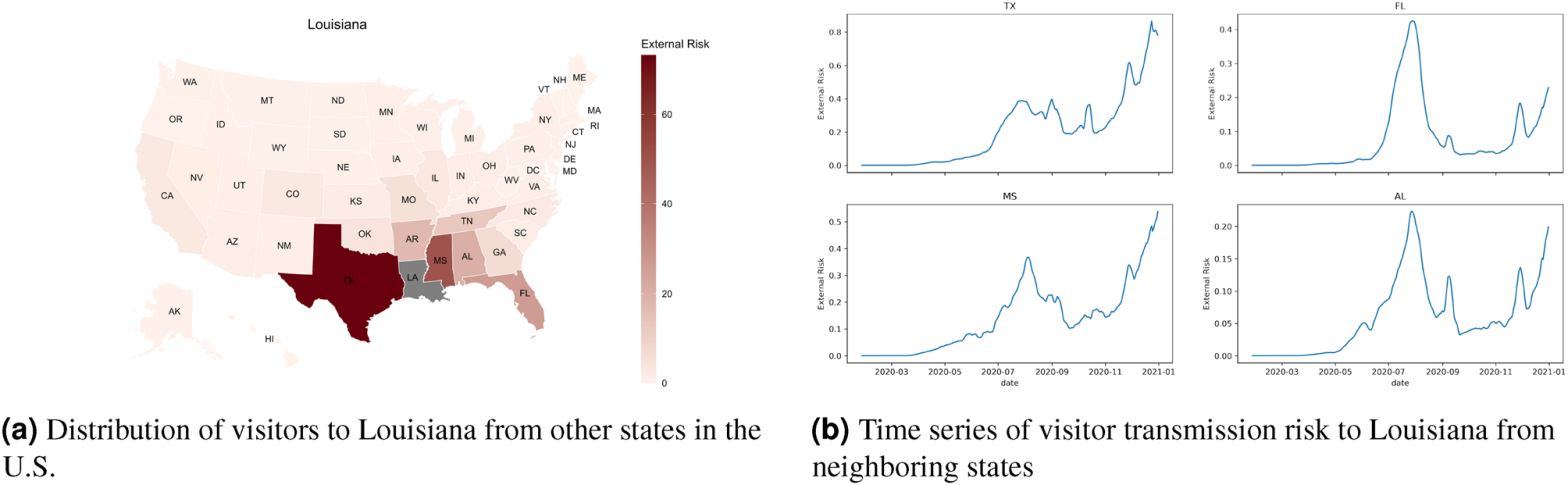
Visitor transmission risk and mobility patterns to the state of Louisiana from other states in the United States. Map generated using urbnmapr^33^.

**Figure 4:**
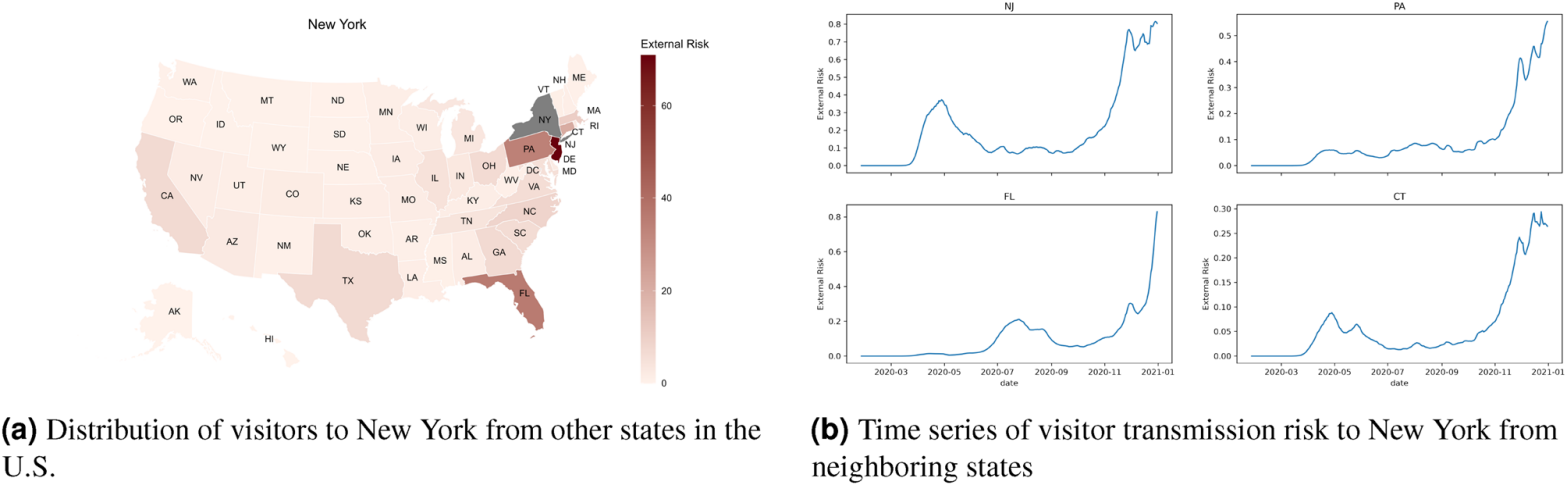
Visitor transmission risk and mobility patterns to the state of New York from other states in the United States. Map generated using urbnmapr^33^.

**Figure 5:**
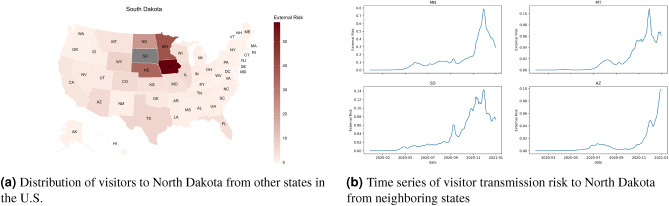
Visitor transmission risk and mobility patterns to the state of North Dakota from other states in the United States. Map generated using urbnmapr^33^.

While it is apparent that the majority of the visitor transmission risk is due to travelers crossing state boundaries from neighboring cities, there is also considerable transmission risk due to long-distance travel. For example, for Louisiana, the majority of the risk for its second peak is contributed by Mississippi, Texas, and Florida, which is higher than the Arkansas that borders the state in the north. The states of Mississippi and Florida contributed more to the second peak, whereas travelers from Texas contributed to the second and third phases of the pandemic. For the states like North Dakota, most of the visitor transmission risk is attributed to their neighboring states. New York, on the other hand, has a huge increase in visitor transmission risk from Florida during the late winter when the state of Florida opened up to travelers compared to the rest of the country. These trends are presented in Figs. 3, 4 and 5.

**Figure 6:**
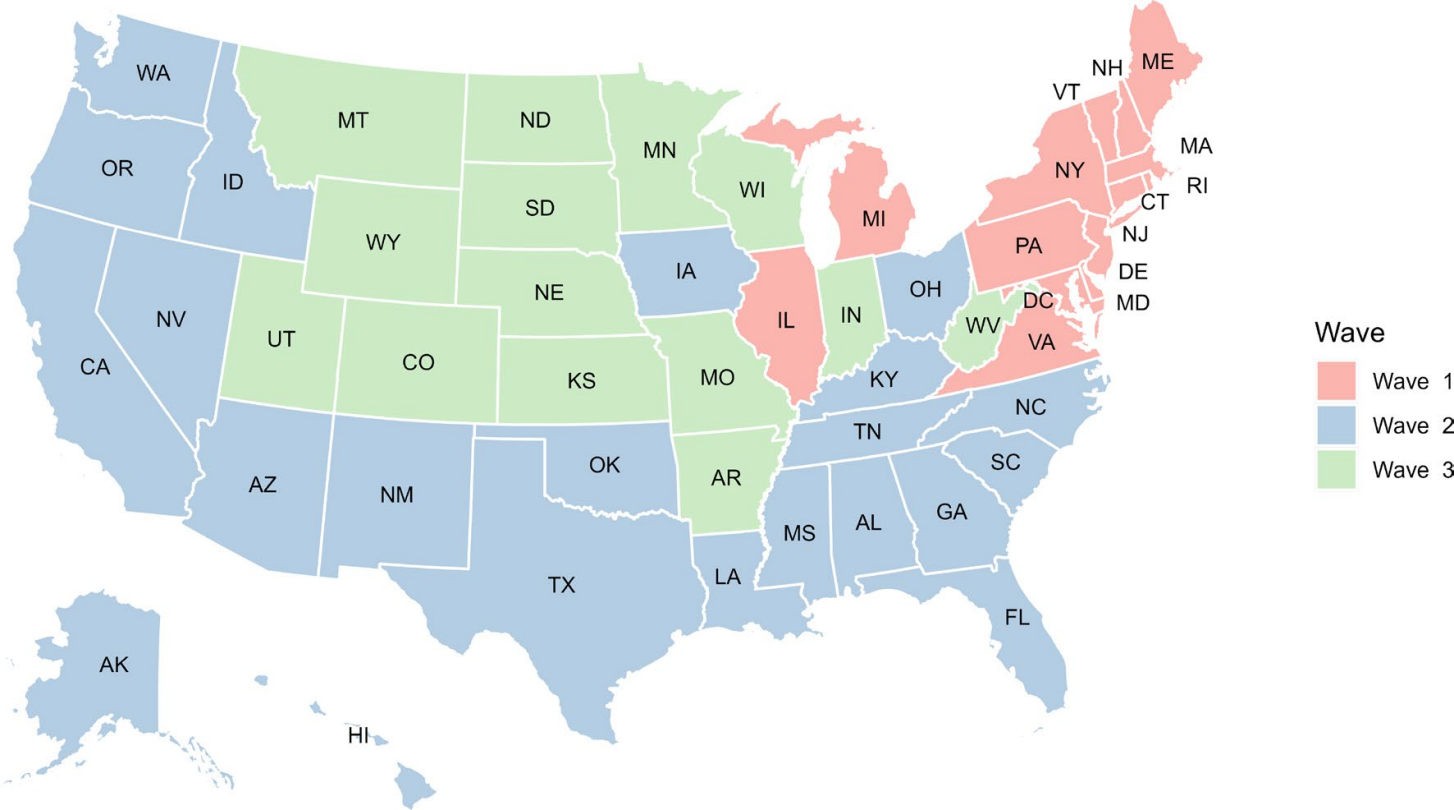
The waves of the pandemic across the United States. Map generated using urbnmapr^33^.

Figure 6 also shows how the pandemic spread across the United States during the first year of the pandemic. The first set of states that saw a significant increase in the number of cases are primarily located in the northeast United States (New England and the tri-state area). Based on Fig. 4, this risk is imported from the neighboring states, i.e., New Jersey and Connecticut, in the case of New York. While all the states restricted travel during this time period, we noticed that interstate travel still contributed a significant risk across these states compared to the rest of the states in the country. During the second wave of the pandemic during the summer, the majority of the states in the southern United States and the West Coast were impacted. The mobility of the individuals is comparatively higher than during the first wave. We also observe that these states had relaxed travel restrictions compared to the states that had a peak during the first wave. Finally, the states that had an increase in the number of cases during the third wave did not have an earlier peak and had a significant increase in mobility, both locally and from outside the states. We would also point out that these states had a higher number of cases in waves 1 and 2, and also had a greater increase in the number of cases in December. The states highlighted in wave 3 had an early rise in cases compared to the rest of the country.

## Limitations and future work

In this study, we explored the impact of local and visitor mobility on the transmission of COVID-19 in all 50 states in the United States. However, here are some areas where this work can be potentially extended. First, the primary objective of this work is to evaluate the impact of internal and external mobility, and their effects on disease incidence. This study does not consider the many mitigating factors imposed by local authorities to curb the spread of the virus; these include: mask usage, lockdowns, social distancing guidelines, and public compliance with health regulations that could have had an effect on the number of cases. The impact of these measures are studied in other works on COVID^34^. Second, the models have been generated on the data aggregated at a daily level of granularity. There have been several issues with the data reported by state and local authorities, that include less testing over the weekend and bulk reporting of missed cases. We handle this problem by smoothening the data over 7 days. Third, the mobility data considers the number of individuals traveling from one state to another but does not capture the distance traveled by individuals during the trip. Incorporating the distance traveled might help enhance the relationship between the number of cases and the mobility of individuals. Fourth, the goal of our excercise is to evaluate the impact of local and visitor mobility on the number of cases; the provided solution is not a forecasting solution to predict the future number of COVID cases. There have been various studies that developed models that take into account: the number of cases, mobility, socio-demographic information, serological impacts etc. to predict the number of cases. The COVID Hub Ensemble model had a MAPE of 11.8% for a 2 week ahead forecast during the first year of the pandemic, DeepCOVID model from Rodriguez et al. had an accuracy of 9.2% during the same time period, compared to 16.8% in our analysis^35,36^. Finally, we consider the state as a single unit to measure the mobility and the number of cases. We do not consider the population density at origin and destination and the number of people traveling to a particular city in a state. For example, the first wave (March–June) was dominated by cases from metropolitan areas, whereas the cases during the third wave were primarily in the rural areas of the state. In the future, we would like to extend this model to various metropolitan areas in the county for analysis at a more refined level of granularity.

## Conclusions

In this paper, we evaluated the impact of the disease transmission risk due to visitor and local mobility on the number of cases at a state level for all 50 states in the United States. We observed that visitor mobility is an important factor in explaining case growth. The prediction accuracy improved by 33.78% for the whole duration of the pandemic in 2020 (March–December) when visitor mobility was used in the forecasting model. The impact of transmission risk due to external mobility is observed across all three phases of the pandemic in the United States. We observe the influence of mobility is much stronger in the first phase of the pandemic compared to the second or third phase. These observations are consistent with some of the earlier studies^4,11^ where mobility was observed to be an important predictor of case growth in the first phase of the pandemic.

## Data Availability

The analysis code for this paper is available on GitHub at https://github.com/raviteja-bhupatiraju/CovidDifferentialMobilityAnalysis.
